# Are Urban Home Health Agencies More Likely to Achieve Improvement in Care Quality?: A Cohort Study

**DOI:** 10.1093/geroni/igaa057.798

**Published:** 2020-12-16

**Authors:** Chenjuan Ma

**Affiliations:** New York University, New York, New York, United States

## Abstract

Home health care (HHC) is a core source of home- and community-based services to older adults “aging in place.’ HHC quality is under increasing scrutiny. This study aimed to examine urban vs. rural disparities in HHC quality in the US. This is a cohort study using 2014-2019 national Home Health Compare data linked to Providers of Services (POS) files. Quality of HHC was measured by agency rates of 1) timely initiation of care and 2) hospitalization. We examined 6,448 home health agencies or 38,688 agency-years. At baseline, the mean rate of timely initiation of care was 91.3% (SD: 7.6; range: 28.0-100.0), with urban agencies performing worse (urban vs. rural: 91.0% vs. 92.3%); and the mean rate of hospitalization was 15.6% (SD: 3.7; range: 0.0-37.0), with urban agencies performing better (urban vs. rural: 15.3% vs. 16.4%). Estimates from multivariate hierarchical linear regressions showed that while the rates of timely initiation of care increased annually over time (β= 0.62, p=0.000), this improvement trends did not differentiate between urban and rural agencies (urban: β= 0.087, p=0.086). There was slight annual improvement in hospitalization rates over time (β= -0.07, p=0.0.003) and the trends in annual reduction in hospitalization rates were more significant in rural agencies than urban agencies (urban: β= 0.10, p=0.000). Our findings indicate urban (vs. rural) disparities in HHC quality and the trends of quality improvement.

